# Ethics Versus Success? The Acceptance of Unethical Leadership in the 2016 US Presidential Elections

**DOI:** 10.3389/fpsyg.2019.03089

**Published:** 2020-01-22

**Authors:** Catarina Morais, Dominic Abrams, Georgina Randsley de Moura

**Affiliations:** ^1^Research Centre for Human Development, Faculty of Education and Psychology, Universidade Católica Portuguesa, Porto, Portugal; ^2^Centre for the Study of Group Processes, School of Psychology, University of Kent, Canterbury, United Kingdom

**Keywords:** unethical, leadership, causal attribution, group processes, election

## Abstract

Before and after the 2016 US Presidential Election, this research examined Trump and Clinton supporters’ attributions about behavior of each leader, both of whose ethicality had been publicly questioned. American voters (*N* = 268) attributed significantly more dispositional factors to the outgroup leader than to the ingroup leader. Moreover, when the ingroup candidate won the election (i.e., among Trump supporters), unethical leadership subsequently became more acceptable and there was less desire to tighten the election process when dealing with unethical candidates. The opposite pattern was found among voters whose ingroup candidate lost the election (Clinton supporters). The results and implications are discussed.

## Introduction

Individuals have a basic need to understand others to ensure efficient social interaction and exchange. This social understanding is achieved by knowing *why* people do what they do: causal knowledge. When searching for causes, people generally resort to processes that require the least cognitive effort, such as reliance on heuristics and stereotypes, to judge and categorize others’ behaviors (Kahneman, 2003). As such, individuals spontaneously infer the causal locus of individuals’ behaviors based on categorization, stereotypes and automatic processing, with causes and consequences of behavior grounded on quickly accessible schemas. A primary question is whether someone’s behavior is the result of their disposition to behave in that particular way or a response to situational constraints (e.g., Kelley and Michela, 1980). It is known that distinctive and consistent behaviors are likely to be attributed to dispositions more than to situations.

Relevant for political campaigns and the present research is the fact that individuals are particularly sensitive to group leaders’ behavior because leaders are distinctive but also central representatives of the group (Haslam et al., 2001; Hogg, 2001; van Knippenberg and van Knippenberg, 2005). Furthermore, during political campaigns group identity and leadership are generally very salient, and candidates’ behaviors are scrutinized closely. The 2016 US Presidential Election was no exception. The main candidates, Donald Trump and Hillary Clinton, were both systematically presented in the media as unethical leaders (e.g., The New York Times, 2015, 2016a,b; The Telegraph, 2017). The present research examines voters’ attributions for leadership candidates’ behavior when both ingroup and outgroup leaders have been portrayed and described as unethical, and how perceptions of the leaders may depend on perceivers’ group membership (Democrat, Republican). We were interested in testing how such perceptions change before and after the election, in the light of ingroup success or failure. The central general proposition, tested in this research, is that when unethical leadership is combined with ingroup success, it encourages a subsequent relaxation of ethical standards, both to justify the success and to enable unethical success to continue.

### Ethical and Unethical Leadership

Ethical leadership has been defined as “the demonstration of normatively appropriate conduct through personal actions and interpersonal relationships, and the promotion of such conduct to followers through two-way communication, reinforcement, and decision-making” (Brown et al., 2005, p. 120). This definition encompasses different and important features of ethical leadership, including being a credible role model and taking ethical issues into consideration when making a decision (Neves and Story, 2015).

The ethical leadership framework holds that leaders are frequently perceived as legitimate role models for normative behavior because of their position within an organization or group (e.g., Mayer et al., 2012). Perceived legitimacy likely enables ethical leaders to influence followers’ ethical conduct. Such legitimacy is achieved as a consequence of followers’ perceptions that the leader behaves in a normatively appropriate manner, is honest, and has altruistic rather than selfish motivation (cf. Brown et al., 2005; Brown and Treviño, 2006; De Hoogh and Den Hartog, 2008).

The ethical leadership framework is consistent with the social identity theory of leadership (Hogg, 2001), which argues that the fundamental mechanism of leadership is the leader’s ability to embody the normative prototype, i.e., the cognitive representation of the characteristics of the group (e.g., Hogg, 2001; Rast, 2015). Therefore, the more prototypical an individual group member is, the more likely that individual is to emerge as the leader because they are viewed by members as best representing the identity of the group (Hogg, 2001; Hogg et al., 2012).

One reason why ingroup and outgroup unethical leaders receive different reactions to their behaviors is because a negative evaluation of a central member of one’s own group conflicts with the need to maintain a positive social identity (Marques et al., 2001; Pinto et al., 2010; Randsley de Moura et al., 2011; Abrams et al., 2013).

Expressing disapproval to an unethical leader may reflect an effort to exert social control over that deviant (cf. Brauer and Chekroun, 2005; Chekroun, 2008), but communicating disapproval directly to the leader may not be feasible, particularly if the group is very large. Previous research has established that ingroup leaders are often given latitude to deviate from group norms (Abrams et al., 2018). Ingroup leaders who commit unethical actions are less likely to be derogated or punished than other ingroup members or outgroup leaders and members who commit the same transgressive actions; a phenomenon labeled ‘transgression credit’ (Abrams et al., 2013; Randsley de Moura and Abrams, 2013).

One explanation for transgression credit is that derogating an ingroup leader may be perceived by other ingroup members as acting against the group. Group members’ motivation to preserve the value of the leader and show respect and loyalty for the group therefore inhibits their critical response to their leader’s transgressions (Zdaniuk and Levine, 2001; Abrams et al., 2013). It is also important to note that transgression credit is more likely to be granted under certain conditions, particularly when the transgression is perceived to be for the benefit of the group and not for leaders’ personal interest (Abrams et al., 2013, 2014).

Previous research has also shown that the outcome of an unethical act may impact group members’ willingness to exert social control and even accept the leaders’ transgression. Morton et al. (2007) showed that ingroup success may be also a boundary condition for group members’ acceptance of deviance. They found that, in a political context, if ingroup members perceived that public opinion was against the group, they gave more support to a candidate whose deviance was linked to having a better chance of being elected. Under such circumstances (ingroup failure), group members also tend to seize on opportunities to challenge the legitimacy of the outgroup success (cf. shifting standards model; Biernat and Manis, 1994; Biernat, 2003) and, therefore, may be more willing to invoke moral standards to do so.

### Group Membership and Causal Attributions of Behavior

Attribution theories emphasize people’s tendency to identify dispositional and situational causes for others’ behavior (Gilbert and Malone, 1995), affecting one’s perception of the amount of control that a certain individual has within a specific situation (Nisbett, 1973). The fundamental attribution error (Heider, 1958) is the tendency to make inferences regarding someone’s unique and enduring dispositions based on behaviors that can be fully explained by the context or situation in which they occur (Gilbert and Malone, 1995). Similar effects occur at the group level, as people make stronger dispositional attributions for outgroup members’ behavior than for ingroup members’ behavior (Allison and Messick, 1985), particularly based on the behavior’s outcome for the group. Indeed, the tendency to attribute ingroup success and outgroup failure to internal dispositions (internal characteristics of the group or their members), and ingroup failure and outgroup success to external factors is referred to as the ultimate attribution error (characteristics of the situation; Pettigrew, 1979). For example, Allison and Messick (1985) found that people also tend to assume that a group’s decision-making is influenced by the attitudes of group members, while ignoring the impact of decision-making rules and group norms.

Interestingly, the ultimate attribution error is particularly strong when people judge outgroups and, more specifically, for negative behaviors (Allison and Messick, 1985). Taken together, these findings suggest that dispositional attributions are more likely for outgroup members’ negative behavior. This suggests that leaders’ unethical actions are more likely to be attributed to a disposition of the leader when people judge outgroup leaders than when they judge ingroup leaders, and that this relationship might be strengthened in cases of outgroup success/ingroup failure and weakened in cases of outgroup failure/ingroup success (e.g., victory/loss in elections).

Another way in which group membership affects attributions is that people differentiate less among outgroup members than among ingroup members in their attributions about different group members’ behavior. This is because outgroups are perceived to be more homogeneous (Quattrone and Jones, 1980).

In the present research, we test how group membership affects evaluations and causal attributions when members face the situation of choosing between two reportedly unethical leaders, and how those evaluations and attributions change based on ingroup success. The US Presidential Election of 2016 provided an opportune moment to pursue this research question because the two main candidates had both been perceived to be, and widely reported as, unethical. Thus, we examined how Clinton and Trump supporters evaluated their respective leaders and their judgments and desirability of leaders’ ethicality in general, before and after the election. The preceding review led us to propose several hypotheses:

#### H1 – Transgression Credit

We expect participants to perceive the ingroup unethical leader as (a) more prototypical and (b) to express more confidence that s/he will be a good president than the outgroup unethical leader. Moreover, (c) we predict a transgression credit effect such that participants will evaluate the ingroup unethical leader more positively and as less self-promoting than the outgroup unethical leader, and (d) this effect is likely to be strengthened post-election.

#### H2 – Attributional Bias

(a) Based on the ultimate attribution error, outgroup unethical leaders’ behavior will be attributed more to *dispositional* and *stable* factors than will that of ingroup unethical leaders. We also expect this relationship (b) to be stronger for the candidate that loses the election and weaker for the candidate that wins the election.

#### H3 – Effect of Success

We expect participants to attribute (a) higher *personal control* and lower *external control* to the outgroup unethical leader than to the ingroup unethical leader; (b) and this difference should be stronger if the outgroup unethical leader wins the election, and weaker if the ingroup leader wins the election, because of the value of the outcome of the election to the group.

#### H4 – Outcome Based Shift of Standards

(a) Voters should regard unethical leadership to be more *unacceptable* and they should support measures of *social control* more if the ingroup unethical leader loses the election and (b) the opposite pattern should arise if the ingroup unethical leader wins the election.

## Materials and Methods

### Participants and Design

Participants were recruited using the Amazon’s Mechanical Turk (MTurk). Initially, 770 participants started the survey, but only 549 met the inclusion criteria and, therefore, were eligible and allowed to proceed. From our initial sample of 549, 154 participants were excluded because they failed attention checks (resulting *n* = 395). Exclusion was equally distributed among supporters of both candidates, χ^2^(1) = 2.18, *p* = 0.140. Wave 2 (post-election) was completed by 268 participants (68%; attrition rates did not differ per candidate supported, χ^2^(1) = 0.04, *p* = 0.844, 38% amongst Trump supporters and 37% amongst Clinton supporters). Eight participants were removed because they reported that they had not voted. Thus, our final sample included 260 participants who completed both waves.

Before the election, 61% of participants said that they would vote for Hillary Clinton and 39% for Donald Trump, but 6% changed their mind between waves. Therefore, in Wave 2, 56% of our sample reported voting for Hillary Clinton (*n* = 146), 39% for Donald Trump (*n* = 100), and 5% for a different candidate (*n* = 14).

Participants (122 males, 138 females) were aged between 19 and 75 years old (*M* = 43.91, *SD* = 13.46). Participant gender was not significantly related to candidate voted for (χ^2^ = 4.66, *p* = 0.097), and as such was not considered further as a factor. Participants all reported being American, and 79% as employed at the time. The majority of participants indicated they were White (85%), followed by Asian (5%), Hispanic (5%), African American (2%), other race (2%), and Mixed race (1%).

The study employed a 2 (Supported candidate: Donald Trump vs. Hillary Clinton) × 2 (Judged candidate: Donald Trump vs. Hillary Clinton) × 2 (Wave: Pre-election vs. Post-election) mixed design, with Supported candidate as a between-participants factor, and Judged candidate and Wave as within-participants’ factors. We will refer to *ingroup condition* when Clinton Supporters judge Hillary Clinton and when Trump Supporters judge Donald Trump, and the *outgroup condition* when participants made judgments about the opposing candidate (Clinton Voters-Donald Trump; Trump Voters-Hillary Clinton).

### Procedure

In order to achieve a demographically diverse and geographically dispersed sample and to collect data in a short period of time (cf. Kees et al., 2017), and also to avoid an effect of major events in the campaign, participants were recruited via Amazon’s MTurk 1 week either side of the election. Before the election (Wave 1, pre-election), participants were asked to complete an online study on Qualtrics about their perceptions regarding the 2016 US Presidential Election. Participants first answered four pre-screening questions/inclusion criteria. The survey only continued if participants indicated they were (1) eligible voters, (2) Americans, (3) had an intention to vote in the election (participants who did not wanted to vote or that had voted absentee were excluded), and (4) intended to vote either for Donald Trump or for Hillary Clinton – the two target candidates for this study.

Using the software TurkPrime, those who participated in W1 (Pre-election) were contacted via email and asked to complete the W2 (Post-election), a week after the election. We expected up to 50% retention rate and power analysis suggested we therefore require a final sample of 166 participants to achieve 90% power in a 2 (between) × 2 × 2 (within) design. We therefore oversampled initially. According to sensitivity analysis, this sample size (246) provided 90% power (α = 0.05) to detect an effect size as small as 0.294 (η_p_^2^ = 0.021) for main effects of supported candidate, 0.180 (η_p_^2^ = 0.008) for main effects of judged candidate and wave (assuming a correlation of 0.50 for the repeated measures), and 0.180 (η_p_^2^ = 0.008) for the between-within interactions (Supported Candidate × Judged Candidate; Supported Candidate × Wave).

### Measures

#### Control Measures

To control for differences between Trump and Clinton supporters’ pre-voting conceptions about their preferred candidate’s ethicality, national identification, interest in the election, and confidence in their candidate, we included the following measures:

##### Perceived ethicality

To measure general preconceptions about the two candidates we adapted the Ethical Leadership Scale (Brown et al., 2005), by asking participants to imagine their preferred candidate as President of the United States, and to rate their agreement (*1* = *strongly disagree, 7* = *strongly agree*) with 10 statements concerning the prospective conduct of that candidate in the White House with other employees (e.g., “Sets an example of how to do things the right way in terms of ethics”). The average of their responses was computed into a global score of perceived ethicality for pre-election (W1, α = 0.98) and post-election (W2, α = 0.99).

##### National identification

Participants rated their agreement with six statements (e.g., “I am proud to be an American”; *1* = *strongly disagree, 7* = *strongly agree*). The items were adapted from Duriez et al. (2013). A national identification score was computed for both waves (α = 0.96, α = 0.95, respectively) based on the average of responses.

##### Electoral interest

Before the election (W1), and adapted from Bølstad et al. (2013), participants were asked nine questions regarding their level of interest in the election (e.g., “How interested are you in the Presidential Election”; *1* = *not at all, 7* = *very interested*), and their voting habits (e.g., “Do you usually vote on Presidential elections?”; *1* = *never, 7* = *always*). A single score was computed by averaging participants’ responses (α = 0.88).

#### Dependent Variables

##### American prototypicality

Participants rated their agreement with five statements (e.g., “Donald Trump [Hillary Clinton] is a good example of the kind of people Americans are,” *1* = *strongly disagree, 7* = *strongly agree*) adapted from Platow and van Knippenberg (2001). Responses were averaged to form a prototypicality score (W1, α = 0.97; W2, α = 0.98).

##### Confidence in the candidate

Before the election (W1), participants were asked “How confident are you that Donald Trump [Hillary Clinton] will be a good President?” (*0–100*).

##### Evaluation

Participants rated the candidates (*1* = *strongly disagree, 7* = *strongly agree*) on 15 traits of warmth and competence (e.g., “trustworthy,” adapted from Fiske et al., 2002; Cuddy et al., 2004). A principal component analysis with Promax rotation revealed one factor (explaining 82% of variance) and, therefore, these traits were collapsed into a single score of evaluation by averaging the items (W1, α = 0.98; W2, α = 0.99).

##### Self-promoting motivation

Participants classified to what extent they believed Donald Trump [Hillary Clinton] ran for Presidency thinking about “the best interests of Americans as a whole” and “his [her] own best interests” (*0–100*). A Motivation Score was created by subtracting one item from the other so positive scores refer to group-serving motivation, and negative scores to self-serving motivation.

##### Causal attributions

Participants were asked to think about the behavior of the candidates and to provide as many reasons as they could think (maximum of 5) for why the candidates exhibited that behavior. After writing the reasons, and using a 9-point bipolar scale, we adapted the Causal Dimension Scale (McAuley et al., 1992), asking participants to rate their perceptions of the causes of candidates’ behavior on four dimensions: locus of causality, personal control, external control, and stability. A principal component analysis with Promax rotation confirmed the four dimensions of the original scale: (1) *Locus of causality* (e.g., “Reflects an aspect of the self – of the situation,” explaining 33% of variance; W1, α = 0.85; W2, α = 0.85), (2) *Personal control* (e.g., “Over which Donald Trump [Hillary Clinton] has power – has no power,” explaining 19% of variance; W1, α = 0.84; W2, α = 0.88), (3) *External control* (e.g., “Over which others have control – have no control,” explaining 11% of variance; W1, α = 0.79; W2, α = 0.83), and (4) *Stability* (e.g., “Permanent – Temporary,” explaining 9% of variance; W1, α = 0.59; W2, α = 0.69). For each dimension, participants’ scores were computed by averaging their responses on the different items. Therefore, lower levels in the scale indicate a more dispositional locus, higher personal control, higher external control, and more stability.

##### Acceptability of unethical leadership

Participants rated on a 7-point scale (*1* = *not at all, 7* = *extremely*) how “acceptable,” “good,” “adequate,” “justifiable” and “tolerable” it is to elect an unethical leader [in general, not specific to their candidate]. A principal component analysis with Promax rotation revealed one factor (explaining 83% of variance). A single score of acceptability of unethical leadership was computed by averaging participants’ responses (W1, α = 0.95; W2, α = 0.97).

##### Election process adjustment (EPA)

Participants rated their agreement (*1* = *strongly disagree, 7* = *strongly agree*) with seven statements regarding hypothetical group actions to exert more social control. A principal component analysis with Promax rotation revealed two factors: (1) *Stricter process* (e.g., “The election process should make it more difficult for someone to become a presidential candidate”; W1, α = 0.77; W2, α = 0.85, explaining 35% of variance); and (2) *Tolerance of criminality* (e.g., “The election process should allow people with criminal records to be candidates”; α = 0.51; W2, α = 0.57, explaining 18% of variance). Participants responses were averaged to compute a score for each dimension.

## Results

### Control Measures

#### Perceived Ethicality

A Supported candidate × Judged candidate × Wave mixed ANOVA revealed non-significant main effects of Supported candidate, *F*(1,235) = 0.20, *p* = 0.655, and Judged candidate, *F*(1,235) = 1.09, *p* = 0.298. Both candidates were perceived as unethical (*M* = 3.45, *SD* = 0.76), as perceived ethicality of both candidates was tested against the scale midpoint (4): Donald Trump (*M* = 3.13, *SD* = 1.99) was perceived as unethical: *t*(259) = 12.87, *p* < 0.001, CI [−1.00, −0.74], whilst this perception was only marginal for Hillary Clinton (*M* = 3.77, *SD* = 1.99): *t*(259) = 1.86, *p* = 0.064, CI [−0.47, 0.01].

A significant main effect of Wave showed that candidates were considered to be less unethical after (*M* = 3.35, *SD* = 0.77) the election than before (*M* = 3.55, *SD* = 0.75), *F*(1,235) = 34.12, *p* < 0.001, η_p_^2^ = 0.127. A significant Supported candidate × Judged candidate interaction, *F*(1,235) = 805.47, *p* < 0.001, η_p_^2^ = 0.774, showed that Trump supporters evaluated him as more ethical than Hillary Clinton [*t*(99) = 18.42, *p* < 0.001, *g* = 2.80, CI [2.41, 3.19]; *M* = 5.06, *SD* = 1.32; *M* = 1.87, *SD* = 0.91, respectively] whereas Clinton supporters evaluated her as more ethical than Donald Trump (*M* = 5.11, *SD* = 1.29; *M* = 1.71, *SD* = 1.00, respectively), *t*(145) = 21.93, *p* < 0.001, *g* = −2.87, CI [−3.21, −2.53]. No other interactions were significant (all *F*s ≤ 1.07, *p* ≥ 0.302).

#### National Identification

A Supported candidate × Wave mixed ANOVA revealed significant main effects of Supported candidate, *F*(1,233) = 52.13, *p* < 0.001, η_p_^2^ = 0.183, and Wave, *F*(1,233) = 6.28, *p* = 0.013, η_p_^2^ = 0.026. Trump supporters reported higher identification with being an American (*M* = 6.04, *SD* = 1.18) than Clinton supporters (*M* = 4.91, *SD* = 1.23); and, overall, participants were more identified with their country before the election (*M* = 5.49, *SD* = 1.31) than after (*M* = 5.28, *SD* = 1.49). There was a significant Supported candidate × Wave interaction, *F*(1,233) = 27.389, *p* < 0.001, η_p_^2^ = 0.105. Simple effects tests showed that Trump supporters’ identification increased from pre-election (*M* = 5.95, *SD* = 1.26) to post-election (*M* = 6.13, *SD* = 1.04), *t*(99) = 2.02, *p* = 0.047, *g* = −0.16, CI [−0.43, 0.12]; and the opposite pattern was revealed for Clinton supporters (*M* = 5.16, *SD* = 1.25; *M* = 4.66, *SD* = 1.47, respectively), *t*(134) = 5.53, *p* < 0.001, *g* = 0.37, CI [0.13, 0.60].

#### Electoral Interest

No factors significantly affected electoral interest. A one-sample *t*-test comparing with the scale midpoint revealed that overall participants were interested in the election (*M* = 5.69, *SD* = 1.09) regardless of whom they voted for, *t*(244) = −1.27, *p* = 0.204.

### Dependent Variables

A Supported candidate × Judged candidate × Wave mixed ANCOVA was conducted for the dependent variables, with Supported candidate as the between-participants factor, Judged candidate and Wave as within-participants, and perceived ethicality (for each candidate and wave) and national identification as covariates. Participants’ identification with the country was included as a covariate because previous research has shown that participants’ identification is an important factor when evaluating group members (e.g., Hutchison and Abrams, 2003). Moreover, we also wanted to ensure that any differences on candidates’ perceived ethicality were not driving the effects. Thus, candidates’ ethicality was controlled for and included as a covariate. The electoral interest variable was not included as covariate because the analysis did not yield any significant differences. Means and standard deviations within and across the design are shown in Table 1.

**TABLE 1:** Means and standard deviations for all measures.

**Variable**	**Voters**	**Candidate: Donald Trump**			**Candidate: Hillary Clinton**			**Total**	
		**W1, *M* (*SD*)**	**W2, *M* (*SD*)**	**Total, *M* (*SD*)**	**W1, *M* (*SD*)**	**W2, *M* (*SD*)**	**Total, *M* (*SD*)**	**W1, *M* (*SD*)**	**W2, *M* (*SD*)**
Prototypicality	Trump	4.83(1.56)	5.12 (1.43)	4.97 (1.17)	1.65 (1.02)	1.77 (0.99)	1.71 (1.20)	3.24 (0.81)	3.45 (0.88)
	Clinton	1.69 (0.88)	1.93 (1.25)	1.81 (1.17)	4.78 (1.41)	4.77 (1.55)	4.78 (1.19)	3.23 (0.81)	3.35 (0.88)
	Total	3.01 (1.97)	3.28 (2.06)	3.39 (1.19)	3.46 (2.00)	3.51 (2.00)	3.24 (1.22)	3.24 (0.82)	3.40 (0.89)

Confidence in	Trump			72.02 (25.31)			10.52 (14.74)		
the candidate	Clinton			7.30 (13.78)			77.88 (21.44)		
	Total			32.90 (37.04)			49.84 (37.52)		

Evaluation	Trump	4.90 (1.45)	5.22 (1.28)	5.06 (1.11)	1.78 (0.92)	1.98 (1.07)	1.88 (1.15)	3.34 (0.74)	3.60 (0.75)
	Clinton	1.66 (0.91)	1.77 (1.02)	1.71 (1.11)	5.08 (1.33)	5.21 (1.36)	5.14 (1.15)	3.37 (0.74)	3.49 (0.75)
	Total	3.03 (1.99)	3.23 (2.05)	3.39 (1.12)	3.68 (2.01)	3.85 (2.03)	3.51 (1.17)	3.35 (0.74)	3.55 (0.75)

Self-promoting	Trump	25.63 (48.66)	41.41 (44.69)	33.52 (38.12)	−73.65(34.56)	−71.98(33.39)	−72.82(40.03)	−24.01(28.89)	−15.29(25.97)
Motivation	Clinton	−75.08(36.65)	−73.76(38.76)	−74.42(38.12)	24.91 (49.90)	27.06 (46.83)	25.99 (40.03)	−25.08(28.89)	−23.35(25.97)
	Total	−32.59(65.21)	−25.16(70.37)	−20.45(38.64)	−16.68(65.69)	−14.73(64.29)	−23.42(40.49)	−24.55(29.25)	−19.32(26.33)

Locus of	Trump	3.21 (1.96)	2.98 (1.85)	3.10 (1.34)	2.22 (1.56)	2.51 (2.00)	2.36 (1.47)	2.71 (1.34)	2.75 (1.35)
causality	Clinton	2.08 (1.48)	1.91 (1.42)	1.99 (1.77)	3.35 (1.78)	3.02 (1.59)	3.18 (1.92)	2.72 (1.33)	2.46 (1.35)
	Total	2.56 (1.78)	2.36 (1.70)	2.55 (1.35)	2.87 (1.78)	2.80 (1.79)	2.77 (1.49)	2.71 (1.35)	2.60 (1.37)

Personal	Trump	2.78 (1.48)	2.56 (1.61)	2.67 (1.97)	3.39 (2.34)	3.18 (2.34)	3.29 (1.66)	3.08 (1.54)	2.87 (1.67)
control	Clinton	4.03 (2.61)	3.65 (2.66)	3.84 (1.98)	2.97 (1.75)	2.69 (1.53)	2.83 (1.66)	3.50 (1.53)	3.17 (1.67)
	Total	3.50 (2.28)	3.19 (2.34)	3.26 (2.00)	3.14 (2.03)	2.90 (1.93)	3.06 (1.68)	3.29 (1.55)	3.02 (1.69)

External control	Trump	6.37 (1.91)	6.80 (1.89)	6.59 (1.58)	6.56 (2.35)	6.10 (2.52)	6.33 (1.79)	6.89 (1.25)	7.12 (1.27)
	Clinton	7.41 (1.89)	7.44 (1.95)	7.42 (1.58)	6.16 (1.89)	6.25 (1.97)	6.20 (1.79)	6.36 (1.62)	6.18 (1.71)
	Total	6.97 (1.96)	7.17 (1.95)	7.01 (1.60)	6.32 (2.10)	6.19 (2.21)	6.27 (1.82)	6.62 (1.59)	6.65 (1.54)

Stability	Trump	4.50 (1.87)	4.30 (1.91)	4.40 (1.69)	3.37 (1.67)	3.89 (2.03)	3.63 (1.55)	3.94 (1.48)	4.10 (1.53)
	Clinton	3.66 (1.97)	4.19(2.26	3.93 (1.70)	4.20 (1.86)	3.97 (1.80)	4.09 (1.55)	3.93 (1.47)	4.08 (1.53)
	Total	4.01 (1.97)	4.24 (2.12)	4.16 (1.71)	3.85 (1.83)	3.94 (1.89)	3.86 (1.57)	3.93 (1.49)	4.09 (1.55)

Acceptability of	Trump							2.04 (1.41)	2.63 (1.67)
unethical	Clinton							1.77 (1.11)	1.47 (0.92)
leadership	Total							1.88 (1.25)	1.97 (1.41)

EPA: Stricter	Trump							4.75 (1.43)	4.13 (1.57)
process	Clinton							4.55 (1.27)	5.11 (1.24)
	Total							4.62 (1.34)	4.70 (1.47)

EPA: Tolerance	Trump							2.08 (1.51)	2.29 (1.60)
of criminality	Clinton							2.96 (1.63)	2.64 (1.59)
	Total							2.58 (1.64)	2.49 (1.60)

A table of ANOVA statistics without covariates is provided (Table 2). In describing the results in the main text, we focus on analyses with covariates included. This is in order to pursue a more conservative presentation of the findings. In almost all cases where effects with inclusion of covariates were *p* < 0.05, the effect is stronger and the significance level smaller when analyses were conducted without covariates. Where findings with 0.10 > *p* > 0.05, inclusive of covariates, are referred to as ‘marginal effects,’ these are all significant at *p* < 0.05 if covariates are not included. If findings were *p* > 0.05 in both analyses, the findings are described as non-significant. Only a few effects were significant solely in the absence of covariates and *p* > 0.10 with the inclusion of covariates. These are noted individually.

**TABLE 2:** ANOVA results for all variables without covariates included.

	**Voters main effect**	**Candidate main effect**	**Wave main effect**	**Voters × Candidate**	**Voters × Wave**	**Candidate × Wave**	**Voters × Candidate × Wave**
American Prototypicality	*F*(1,233) = 0.20, *p* = 0.652	*F*(1,233) = 2.18, *p* = 0.141	*F*(1,233) = 14.31, *p* < 0.001, η_p_^2^ = 0.058	*F*(1,233) = 711.50, *p* < 0.001, η_p_^2^ = 0.753	*F*(1,233) = 1.36, *p* = 0.244	*F*(1,233) = 3.51, *p* = 0.062, η_p_^2^ = 0.015	*F*(1,233) = 0.40, *p* = 0.529
Confidence in the candidate	*F*(1,242) = 0.02, *p* = 0.885	*F*(1,242) = 5.30, *p* = 0.022, η_p_^2^ = 0.021		*F*(1,242) = 1178.92, *p* < 0.001, η_p_^2^ = 0.830			
Evaluation	*F*(1,233) = 0.89, *p* = 0.346	*F*(1,233) = 7.34, *p* = 0.007, η_p_^2^ = 0.031	*F*(1,233) = 16.61, *p* < 0.001, η_p_^2^ = 0.067	*F*(1,233) = 947.84, *p* < 0.001, η_p_^2^ = 0.803	*F*(1,233) = 4.15, *p* = 0.043, η_p_^2^ = 0.017	*F*(1,233) = 0.39, *p* = 532	*F*(1,233) = 0.81, *p* = 0.366
Self-promoting Motivation	*F*(1,233) = 1.95, *p* = 0.164	*F*(1,233) = 1.14, *p* = 0.288	*F*(1,233) = 11.40, *p* = 0.001, η_p_^2^ = 0.047	*F*(1,233) = 705.68, *p* < 0.001, η_p_^2^ = 0.752	*F*(1,233) = 5.10, *p* = 0.025, η_p_^2^ = 0.021	*F*(1,233) = 4.59, *p* = 0.033, η_p_^2^ = 0.019	*F*(1,233) = 5.84, *p* = 0.016, η_p_^2^ = 0.024
Locus of causality	*F*(1,233) = 0.79, *p* = 0.376	*F*(1,233) = 11.16, *p* = 0.046, η_p_^2^ = 0.017	*F*(1,233) = 1.24, *p* = 0.266, η_p_^2^ = 0.005	*F*(1,233) = 77.21, *p* < 0.001, η_p_^2^ = 0.249	*F*(1,233) = 2.44, *p* = 0.119	*F*(1,233) = 1.44, *p* = 0.231	*F*(1,233) = 4.55, *p* = 0.034, η_p_^2^ = 0.019
Personal control	*F*(1,233) = 3.87, *p* = 0.050, η_p_^2^ = 0.016	*F*(1,233) = 1.48, *p* = 0.225, η_p_^2^ = 0.006	*F*(1,233) = 6.37, *p* = 0.012, η_p_^2^ = 0.027	*F*(1,233) = 28.97, *p* < 0.001, η_p_^2^ = 0.111	*F*(1,233) = 0.40, *p* = 0.526	*F*(1,233) = 0.09, *p* = 0.770	*F*(1,233) = 0.04, *p* = 0.839
External control	*F*(1,233) = 3.60, *p* = 0.059, η_p_^2^ = 0.015	*F*(1,233) = 29.67, *p* < 0.001, η_p_^2^ = 0.113	*F*(1,233) = 0.01, *p* = 0.960	*F*(1,233) = 12.33, *p* = 0.001, η_p_^2^ = 0.050	*F*(1,233) = 0.30, *p* = 0.591	*F*(1,233) = 3.83, *p* = 0.052, η_p_^2^ = 0.016	*F*(1,233) = 5.18, *p* = 0.024, η_p_^2^ = 0.022
Stability	*F*(1,233) = 0.01, *p* = 0.969	*F*(1,233) = 6.01, *p* = 0.015, η_p_^2^ = 0.025	*F*(1,233) = 2.22, *p* = 0.137	*F*(1,233) = 13.63, *p* < 0.001, η_p_^2^ = 0.055	*F*(1,233) = 0.01, *p* = 0.942	*F*(1,233) = 0.01, *p* = 0.971	*F*(1,233) = 14.34, *p* < 0.001, η_p_^2^ = 0.058
Acceptability of unethical leadership	*F*(1,231) = 22.99, *p* < 0.001, η_p_^2^ = 0.091		*F*(1,231) = 3.64, *p* = 0.058, η_p_^2^ = 0.016		*F*(1,231) = 30.63, *p* < 0.001, η_p_^2^ = 0.117		
EPA: Stricter process	*F*(1,231) = 6.24, *p* = 0.013, η_p_^2^ = 0.026		*F*(1,231) = 0.12, *p* = 0.729		*F*(1,231) = 47.89, *p* < 0.001, η_p_^2^ = 0.172		
EPA: Tolerance of criminality	*F*(1,231) = 11.63, *p* = 0.001, η_p_^2^ = 0.048		*F*(1,231) = 0.29, *p* = 0.592		*F*(1,231) = 7.95, *p* = 0.005, η_p_^2^ = 0.033

Transgression credit: H1 (a) Participants will perceive the ingroup unethical leader as more prototypical and (b) be more confident in their leadership. They will also (c) evaluate the ingroup unethical leader more positively and as less self-promoting than the outgroup unethical leader, and (d) these differences will be stronger post-election.

Regarding *prototypicality*, the marginal main effects of Supported candidate, *F*(225) = 3.27, *p* = 0.072, η_p_^2^ = 0.014, and Judged candidate, *F*(225) = 3.01, *p* = 0.084, η_p_^2^ = 0.013, indicated that Trump supporters tended to rate both candidates as slightly more prototypical (*M* = 3.45, *SD* = 0.88) than Clinton supporters (*M* = 3.20, *SD* = 0.81). Hillary Clinton was perceived to be slightly more prototypically American (*M* = 3.45, *SD* = 0.67) than Donald Trump (*M* = 3.20, *SD* = 0.84).

A significant Supported candidate × Judged candidate interaction, *F*(1,225) = 7.15, *p* = 0.008, η_p_^2^ = 0.031, revealed that prototypicality was perceived to be higher for the candidate that participants supported. The ingroup unethical leader was perceived as more group prototypical than the outgroup unethical leader, supporting H1a. No other main effects or interactions were significant (all *F*s ≤ 3.27, *p* ≥ 0.072).

Regarding *confidence in the candidate*, there was a marginal main effect of Judged candidate, *F*(1,266) = 3.23, *p* = 0.073, η_p_^2^ = 0.012. Participants were slightly more confident that Hillary Clinton would be a good president (*M* = 49.84, *SD* = 37.52) than Donald Trump (*M* = 32.90, *SD* = 37.04). The main effect of Supported candidate was not significant, *F*(1, 266) = 0.12, *p* = 0.914. The significant Supported candidate × Judged candidate interaction, *F*(1,266) = 1184.48, *p* < 0.001, η_p_^2^ = 0.817, showed the expected pattern, namely that participants believed that the ingroup leader would be a better president than the outgroup leader. That is, Trump supporters were more confident in their candidate (ingroup condition) than in Hillary Clinton (outgroup condition), and the same happened for Clinton supporters, who were more confident in her (ingroup condition) than in Donald Trump (outgroup condition), supporting H1b. The main effects were not significant (all *F*s ≤ 3.23, *p* ≥ 0.073).

Regarding *evaluation*, a marginal main effect of Judged candidate showed that overall Donald Trump was evaluated more negatively (*M* = 3.42, *SD* = 0.51) than Hillary Clinton (*M* = 4.15, *SD* = 0.55), *F*(1,225) = 3.61, *p* = 0.059, η_p_^2^ = 0.016. There was also a significant Supported candidate × Judged candidate interaction, *F*(1,225) = 23.84, *p* < 0.001, η_p_^2^ = 0.096. Participants evaluated the ingroup leader more positively than the outgroup leader, supporting H1c.

The Supported candidate × Wave interaction was also significant, *F*(1,225) = 5.83, *p* = 0.017, η_p_^2^ = 0.025, revealing that Trump supporters gave less negative evaluations after the election than before the election (*M* = 3.60, *SD* = 0.75; *M* = 3.34, *SD* = 0.74, respectively; *t*(99) = 3.92, *p* < 0.001, *g* = −0.29, CI [−0.57, −0.01]), regardless of the candidate being evaluated. The pre-post difference was not significant for Clinton supporters, *t*(145) = 1.316, *p* = 0.137. No other main effects or interactions were significant (all *F*s ≤ 2.03, *p* ≥ 0.156) regarding the evaluation of the candidates.

The same pattern arose on the measure of *self-promoting motivation*. The significant Supported candidate × Judged candidate interaction, *F*(1,225) = 8.16, *p* = 0.005, η_p_^2^ = 0.035, indicated that participants perceived the outgroup unethical leader to be more self-serving than the ingroup unethical leader (cf. Table 1). Therefore, H1c was fully supported.

No other main effects or interactions were significant (all *F*s ≤ 3.35, *p* ≥ 0.068). No three-way interaction was found. Therefore, H1d was not supported.

Attributional bias: H2 (a) Behavior of outgroup unethical leaders will be attributed more to dispositional and stable factors than that of ingroup unethical leaders; (b) this relationship will be stronger for the candidate that loses the election and weaker for the candidate that wins the election.

Regarding locus of causality, a significant supported candidate × Judged candidate interaction, *F*(1,225) = 7.61, *p* = 0.006, η_p_^2^ = 0.033, showed participants perceived the behavior of the outgroup unethical leader as more dispositional than the behavior of the ingroup unethical leader. Therefore, H2a was supported for locus of causality. No other main effects or interactions were found for this variable (all *F*s ≤ 2.01, *p* ≥ 0.158), thus, H2b was not confirmed.

H2a and H2b were not supported for stability, because no main effects nor interactions were significant (all *F*s ≤ 2.02, *p* ≥ 0.156).

Effect of success: H3 We expect participants to attribute (a) higher personal control and lower external control to the outgroup unethical leader than to the ingroup unethical leader; (b) and this relationship will be stronger if the outgroup unethical leader wins the election, and weaker if the ingroup leader wins the election.

Regarding personal control, a significant main effect of Wave, *F*(1,225) = 5.41, *p* = 0.021, η_p_^2^ = 0.023, revealed that participants perceived candidates to have more control over their behaviors after the election (*M* = 3.04, *SD* = 1.79) than before (*M* = 3.31, *SD* = 1.62). However, these perceptions did not differ per Candidate; thus, H3b was not supported. No other main effects or interactions were found (all *F*s ≤ 0.26, *p* ≥ 0.609). Therefore, H3a was not supported for this variable.

A marginal Supported candidate x Judged candidate interaction, *F*(1,225) = 3.85, *p* = 0.051, η_p_^2^ = 0.017, revealed that the ingroup leader was perceived as having more *external control* (*M* = 6.13, *SD* = 1.63) than the outgroup leader (*M* = 7.37, *SD* = 1.54). This pattern was significant for Clinton supporters (ingroup *M* = 6.20, *SD* = 1.79; outgroup *M* = 7.42, *SD* = 1.58, *t*(145) = 8.34, *p* < 0.001, *g* = 0.78, CI [0.54, 1.02]), but not for Trump supporters (ingroup *M* = 6.59, *SD* = 1.58; outgroup *M* = 6.33, *SD* = 1.79), *t*(97) = 1.09, *p* = 0.277. Therefore, H3a was partially supported. No other main effects or interactions were significant (all *F*s ≤ 2.88, *p* ≥ 0.091).

Outcome based shift of standards (a) Voters should regard unethical leadership to be more *unacceptable* and they should support measures of *social control* more if the ingroup unethical leader loses the election and (b) the opposite pattern should arise if the ingroup unethical leader wins the election.

Regarding *acceptability of unethical leadership*, a significant main effect of Supported candidate, *F*(1,225) = 7.59, *p* = 0.006, η_p_^2^ = 0.033, indicated Trump supporters found unethical leadership to be less unacceptable (*M* = 2.34, *SD* = 1.11) than did Clinton supporters (*M* = 1.62, *SD* = 1.12). The main effect of Wave was not significant, *F*(1,225) = 0.06, *p* = 0.813.

**FIGURE 1:**
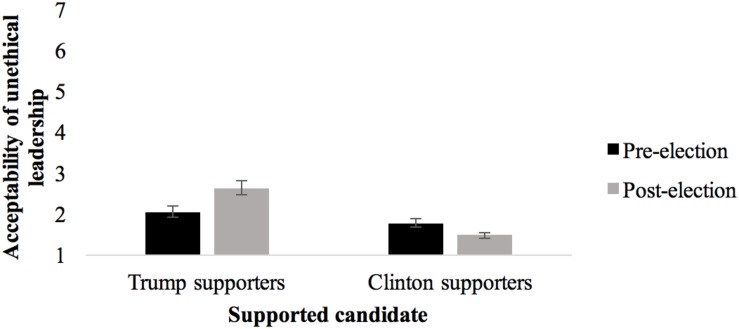
Trump and Clinton supporters’ Acceptance of unethical Leadership Before and After 2016 US Presidential Election (means and standard errors).

Consistent with our hypothesis, a significant Supported candidate × Wave interaction, *F*(1,225) = 8.61, *p* = 0.004, η_p_^2^ = 0.037, showed that when the ingroup unethical leader won the election (Trump supporters), unethical leadership became more acceptable after the election (*M* = 2.63, *SD* = 1.67) than before (*M* = 2.04, *SD* = 1.41), *t*(99) = 3.90, *p* < 0.001, *g* = −0.38, CI [−0.66, −0.10]. The opposite pattern was found amongst the group that lost the election (Clinton supporters). For them, unethical leadership became even more unacceptable after the election (*M* = 1.47, *SD* = 0.92) than before (*M* = 1.77, *SD* = 1.11), *t*(134) = 3.62, *p* < 0.001, *g* = 0.29, CI [0.05, 0.53]. Thus, H4a and H4b were supported for this variable (cf. Figure 1).

**FIGURE 2:**
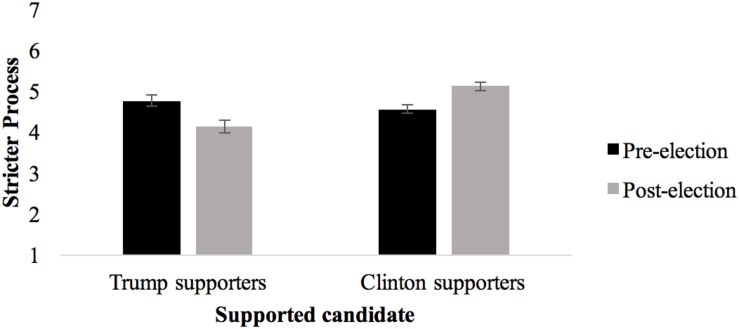
Trump and Clinton supporters’ Acceptance of Stricter Election Before and After 2016 US Presidential Election (means and standard errors).

The election process adjustment (EPA) measure encompassed two dimensions: stricter process and tolerance of criminality. A significant Supported candidate x Wave interaction on stricter process, *F*(1,225) = 10.25, *p* = 0.002, η_p_^2^ = 0.044, showed that Trump supporters became less approving of a strict process following the election (pre*M* = 4.75, *SD* = 1.43; post*M* = 4.13, *SD* = 1.57), *t*(99) = 4.38, *p* < 0.001, *g* = 0.41, CI [0.13, 0.69]. In contrast, Clinton supporters believed that the process should be made stricter post-election (pre*M* = 4.55, *SD* = 1.27; post*M* = 5.11, *SD* = 1.24), *t*(134) = 5.52, *p* < 0.001, *g* = −0.44, CI [−0.69, −0.20]. Therefore, H4a and H4b were fully supported (cf. Figure 2). The main effects were not significant (all *F*s ≤ 1.54, *p* ≥ 0.215).

The significant Supported candidate × Wave interaction on tolerance of criminality revealed a comparable pattern, *F*(1,225) = 4.51, *p* = 0.035, η_p_^2^ = 0.020. Clinton supporters were less tolerant of a leader’s criminal past following the election than they had been before the election (pre*M* = 2.96, *SD* = 1.63; post*M* = 2.64, *SD* = 1.59; *t*(134) = 2.40, *p* = 0.018, *g* = 0.20, CI [−0.04, 0.44]). In contrast, a marginal effect shows that Trump supporters seemed to be more tolerant of leader criminality after the election than before (pre*M* = 2.08, *SD* = 1.51; post*M* = 2.29, *SD* = 1.60), *t*(99) = 1.67, *p* = 0.098, *g* = −0.13, CI [−0.41, 0.14]. The main effects of Voters and Wave were not significant (all *F*s ≤ 1.16, *p* ≥ 0.283).

## Discussion

When facing the situation of choosing or electing major leaders, particularly in the political realm, it is rare that any candidate has an unquestionable record. The question of how people select leaders when the choices are reportedly unethical (and perceived as unethical) has not been addressed by previous research. The present research sheds light on some of the group-related psychological processes that occur when people have to choose between two reportedly unethical leaders. Our central general proposition was that when unethical leadership is combined with ingroup success, it encourages a subsequent relaxation of ethical standards, both to justify the success and to enable unethical success to continue.

Although our sample of voters considered both candidates overall as being low in ethicality and as non-prototypical, they evaluated their ingroup leader more positively, as more ethical, as more prototypical, and as less self-promoting than the outgroup leader. The behavior of the ingroup leader was also perceived as affected more by external/situational (rather than internal/dispositional) factors than was the outgroup leader’s behavior. Moreover, the election result impacted on voters’ acceptance of unethical leadership. When the ingroup leader lost the election, unethical leadership became less acceptable and strengthened the desire for a stricter election process. However, when the ingroup leader won the election, unethical leadership became more acceptable and group members were content to relax the election process. This helps to establish that perception of the acceptability of unethical leadership is dynamic, and not stable over time or context. Moreover, these shifts on attitudes based on ingroup/outgroup success are in line with previous research showing individuals’ needs to maintain balance and to protect their self-esteem by either affiliating more strongly to their group if it is victorious and less so if it loses (cf. BIRGing/CORFing literature, Cialdini et al., 1976; Cialdini and Richardson, 1980).

Overall, the more positive evaluations given to the ingroup leader, when compared to the outgroup leader, are consistent with social identity theory’s assumption that individuals strive to achieve and maintain a positive social identity and, therefore, when engaging in social comparison, they tend to display an ingroup bias (cf. Abrams and Hogg, 1988). Similarly, the fact that the ingroup leader was perceived as being less self-promoting is also consistent with previous findings (e.g., van Knippenberg and van Knippenberg, 2005) which suggest that a leader who displays self-sacrifice communicates the message of being pro-group oriented which, in turn, shows commitment to the collective and attracts stronger support.

In terms of causal attributions, participants perceived the behavior of the outgroup leader to be less affected by external factors and more by the leaders’ internal dispositions, when compared to the behavior of ingroup leaders. This is consistent with Allison and Messick’s (1985) finding that people make stronger dispositional attributions for behaviors by outgroup members than by ingroup members. However, these results did not change according to the election outcome, as we would expect, and an ultimate attribution error did not occur. One possible explanation may be related to the impact of perceptions of ethicality when making attributions, as the Wave effect disappeared when controlling for this measure. Moreover, as leaders occupy a central role within the group, it makes sense that their overall behavior is perceived as stable and as being under high personal and low external control (Hogg et al., 2012). Nevertheless, we expected these perceptions to be affected by participants’ group membership and to depend on the results of the election. Indeed, participants attributed lower external control to the outgroup leader than to ingroup leader, but this result was only verified for Clinton Voters, and did not extend to attributions of stability or personal control.

Based on the present evidence, the 2016 US election results may have had a discernable impact on some supporters’ willingness to accept unethical leadership. The generality of the finding that unethical leadership was more acceptable when the election was won by the ingroup leader and more unacceptable when won by the outgroup leader is informed by consistency with evidence from Morton et al. (2007) experiments. They found that participants were more tolerant of an ingroup deviant political candidate when they perceived the public opinion to be against their group, considering it to be more important that the group achieved its goals (electing their candidate). The present research shows that acceptance of unethical *leadership* in general is also manifested in varying levels of demand for social control. Voters for the winning candidate subsequently advocated a more relaxed electoral process whereas voters for the losing candidate endorsed a stricter election process. Thus, the ingroup benefit of any unethical leader behavior affects not only group members’ endorsement of their leader, but also their willingness to tolerate unethical leadership in future.

These findings have implications for our understanding of system justification or procedural justice processes (Tyler, 1987; cf. Azzi and Jost, 1997; Blasi and Jost, 2006), which could be pursued in future research. It also suggests why it is that leaders who succeed in bending the rules to strengthen their ingroup’s power or achievements seem to be able to attract further support from their electorates (despotic or populist leadership of the major continents and countries such as Turkey spring to mind), despite questions surrounding the propriety of their actions. If voters relax their ethical standards for leadership as a means of bolstering the legitimacy of their ingroup’s success, this could foster abandonment of such standards, allowing the consolidation of increasingly ruthless and unethical governance.

## Conclusion

Taking the US Presidential election as a framework, the present research shows that group members’ perceptions of leadership ethicality may affect behavioral attributions about their leaders, and the acceptability and endorsement of future unethical leadership. This supports the central argument of the present article, that when unethical ingroup leadership succeeds (wins) it encourages both transgression credit and a relaxation of that group’s ethical standards. This potential for leader-driven ethical slippage underlines how important it is that organizations should institute and maintain procedures to hold their leaders to account and to ensure that they uphold scrupulous ethical standards.

Although it would be desirable to find a more ethnically diverse sample as the majority of the present sample (85%) was Caucasian, our sample is not dissimilar from the electorate (cf. Supplementary Material; Pew Research Center, 2018). However, other differences among Clinton and Trump supporters, such as their personality or political ideology, may contribute to the effect of winning/losing on ethicality. Our real-life real-time study has the advantage of high ecological and external validity, dealing with an issue of considerable political and global significance, given the current rise of populism. Moreover, our research suggests that members of victorious groups might conclude that “the ends justify the means,” such that they are more willing to accept the unacceptable in order to gain or retain power for an ingroup leader. Future research could explore, experimentally or longitudinally, possible causal roles of various factors that might increase or attenuate the likelihood that unethical victors will induce relaxation of ethical standards among their supporters, as well as test these effects for different intergroup relations.

## Data Availability Statement

The raw data supporting the conclusions of this article will be made available by the authors, without undue reservation, to any qualified researcher.

## Ethics Statement

The studies involving human participants were reviewed and approved by the University of Kent Ethics Committee. The patients/participants provided their written informed consent to participate in this study.

## Author Contributions

CM and DA conceived the presented idea as part of a research program involving all authors. CM designed the materials and collected and analyzed the data, on which DA and GR provided comments and revisions. All authors discussed the results and associated implications for theory, and contributed to writing the final manuscript.

## Conflict of Interest

The authors declare that the research was conducted in the absence of any commercial or financial relationships that could be construed as a potential conflict of interest.
