# Supplementary material for: Ethics Versus Success? The Acceptance of Unethical Leadership in the 2016 US Presidential Elections
Source: Front Psychol. 2020 Jan 22;10:3089. doi: 10.3389/fpsyg.2019.03089 (PMC6987078; doi:10.3389/fpsyg.2019.03089)
Supplement: Supplementary file 1 [file Table_1.docx]

**Supplementary Material**

*Table 1***.**

*Demographic comparisons among the 2016 valid electorate (cf. Pew Research Center, 2018) and our sample for Clinton and Trump Supporters.*

|  | **Female** | **White** | **High school diploma** | **18-29 years** | **30-49 years** | **50+ years** |
| --- | --- | --- | --- | --- | --- | --- |
| **Electorate** | 55% | 74% | 30% | 13% | 30% | 56% |
| Clinton | 54% | 60% | 28% | 58% | 51% | 45% |
| Trump | 39% | 88% | 34% | 28% | 40% | 52% |
| **Study sample** | 53% | 85% | 29% | 17% | 25% | 59% |
| Clinton | 59% | 82% | 20% | 55% | 58% | 56% |
| Trump | 45% | 92% | 34% | 39% | 36% | 40% |
